# "You can't always get what you want": from doctrine to practicability of study designs for clinical investigation in endometriosis

**DOI:** 10.1186/s12905-015-0248-4

**Published:** 2015-10-22

**Authors:** Paolo Vercellini, Edgardo Somigliana, Ivan Cortinovis, Benedetta Bracco, Lucrezia de Braud, Dhouha Dridi, Silvano Milani

**Affiliations:** Department of Clinical Sciences and Community Health, Università degli Studi and Fondazione Ca’ Granda - Ospedale Maggiore Policlinico, Via Commenda 12, 20122 Milan, Italy; Infertility Unit, Fondazione Ca’ Granda - Ospedale Maggiore Policlinico, Via Manfredo Fanti 6, 20122 Milan, Italy; Unit of Medical Statistics and Biometry, Department of Clinical Sciences and Community Health, Università degli Studi, Via Vanzetti 5, 20133 Milan, Italy

**Keywords:** Endometriosis, Randomized controlled trial, Observational study, Patient preference study, Before and after study

## Abstract

**Background:**

Patients, now generally well informed through dedicated websites and support organizations, are beginning to look askance at clinical experimentation. We conducted a survey investigation to verify whether women with endometriosis would still accept to participate in a randomized controlled trial (RCT) on treatment for pelvic pain.

**Methods:**

A total of 500 patients consecutively self-referring to an academic outpatient endometriosis clinic, were asked to compile two questionnaires focused on hypothetical comparisons between a new drug and a standard drug, and between medical and surgical treatment, for endometriosis-associated pelvic pain. The main outcome measure was the percentage of patients willing to participate in a theoretical RCT.

**Results:**

A total of 239 (48 %) women would decline participation in a comparative study on a new drug and a standard drug, as 204 (41 %) would prefer the former medication, and 35 (7 %) the latter. Fifty women (10 %) would participate in a RCT, but only 24 (5 %) would accept blinding. The most frequently chosen option was the patient preference trial (211; 42 %). No significant differences were observed in demographic and clinical characteristics between the 50 women who would accept and the 450 who would decline to be enrolled in a RCT. A total of 229 women (46 %) would decline participation in a comparative study on medical versus surgical treatment, as 186 (37 %) would prefer pharmacological therapy and 43 (9 %) a surgical procedure. Only 11 (2 %) women would participate in such a RCT. More than half of the women (260; 52 %) selected the patient preference trial. No significant variations in distributions of answers were observed between women who did or did not undergo a previous surgical procedure.

**Conclusion:**

Only a small minority of the women included in our study sample would accept randomization, and even less so blinding. Patient preference appears to play a central role when planning interventional trials on endometriosis-associated pelvic pain. Adequately designed observational analytic studies could be considered when recruitment in a RCT appears cumbersome.

## Background

The randomized controlled trial (RCT) constitutes the gold standard in the hierarchy of studies aimed at evaluating the effect of treatments [1]. In a RCT, only the play of chance and the effect of interventions may explain a difference in outcome, and when chance is not a reasonable explanation, then a variation in treatment effect must be the source of that observed difference [2].

However, several obstacles are rendering gradually more problematic the adoption of this study design [3–8]. Strict regulatory requirements have greatly increased the burden of administrative and bureaucratic duties. Moreover, the costs for protocol approval, trial monitoring, and insurance coverage are steadily rising. In the endometriosis field, this is causing the progressive fading of independent clinical investigation and proliferation of industry-sponsored trials, with potential consequences on both, primary and secondary research [9–11]. In addition, ethical issues on randomization have been described [12].

Also patients, now generally well informed through dedicated websites and support organizations [13], are beginning to look askance at clinical experimentation. Kramer et al. [6] already pointed out the impediments to clinical investigation deriving from reluctance of patients to enroll in trials, and Braunstein et al*.* [14] reported that a great proportion of interviewed trial participants felt that they were likely to be “used” during clinical research. The doubt here is whether, beyond ethical and methodological considerations, women with endometriosis would still easily accept to participate in a RCT.

To answer this question, and to verify if a practical problem of recruitment exists that would create an additional major obstacle to the organization of high-quality clinical research in endometriosis, we conducted a survey investigation on a series of consecutive women self-referring to our outpatient endometriosis clinic.

## Methods

This prospective, descriptive study was conducted in an academic department specializing in the study and treatment of endometriosis and was approved by the local ethics committee (Comitato Etico della Fondazione IRCCS Ca’ Granda Ospedale Maggiore Policlinico, Milano, Italy, approval #247/2013). Italian women with a diagnosis of endometriosis, consecutively self-referring for the first time to our tertiary care outpatient clinic, were asked to participate in a survey to investigate their opinion regarding a potential participation in a hypothetical trial on treatment for pelvic pain symptoms, and to assess their preferences for different study designs. The objective of the survey was to define the percentage of women who would be willing to participate in a randomized, controlled trial.

Women were given written information on the research activity of our center and the reasons for conducting clinical studies. The various conditions amenable to drug therapy and surgery were described. They were told that the quality of study designs is important for the validity of the results and, hence, for the consequent potential benefit for all the patients with endometriosis. Explanations were included on the types of trial that can be planned in order to assess the efficacy of pharmacological therapy or to compare medical with surgical treatment, and they were informed that the most appropriate experimental design is the double-blinded RCT, followed by the open RCT, and finally by the observational analytic studies such as the patient preference trial. Women were invited to ask questions in case of doubts, and the medical personnel of our centre offered immediate clarifications. At the end of the information phase, women who decided to participate in the survey were requested to complete two simple multiple-choice questionnaires on two hypothetical comparisons, one between two drugs and one between medical therapy and surgical treatment, and to sign a written informed consent in which it was stated that the compiled questionnaires would be used for research purposes only.

**Fig. 1:**
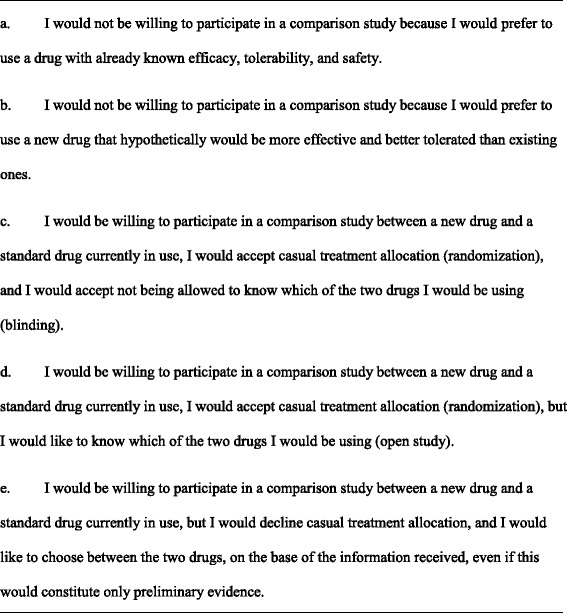
Questionnaire on willingness to participate in a study comparing a new and a standard drug

**Fig. 2:**
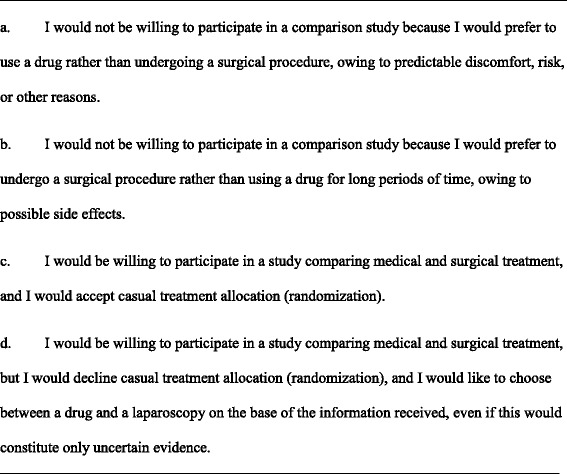
Questionnaire on willingness to participate in a study comparing medical and surgical treatment

The first questionnaire, focused on a hypothetical comparison between a new drug and a standard drug for pelvic pain associated with endometriosis (Fig. 1), and included five different mutually exclusive preferences, i.e., standard drug, new drug, RCT with allocation blinding, RCT without blinding, and patient preference trial. The second questionnaire focused on a hypothetical comparison between a medical and surgical treatment for endometriosis-associated pelvic pain (Fig. 2). Women were invited to imagine that there was uncertainty on which treatment was more effective, and that surgery could be performed at laparoscopy with a risk of around 5 % of mostly minor complications. In addition, they were informed that drugs generally control the disease but do not cure it; on the other side, surgery must often be combined with postoperative medical therapy in order to limit the probability of symptoms and lesions recurrence. In this case, only four mutually exclusive choices were listed, i.e., medical treatment, surgical treatment, RCT, and patient preference trial, as it was deemed that a sham operation (RCT with allocation blinding) would not be easily feasible in our hospital.

Before participants indicated their preferences by circling the chosen alternative on the two questionnaires, they were informed that the study had only a descriptive purpose and that their choices would not imply future obligations or commitments. Collected demographic and clinical information of participants included age, parity, smoking, body mass index, education, previous medical or surgical treatment, and types of medication used. Frequency and severity of dysmenorrhea, deep dyspareunia and non-menstrual pelvic pain were measured according to a 0–3 point multidimensional categorical rating scale devised by Biberoglu and Behrman [15], and also using a 0 to 10 numerical rating scale, with 0 indicating the absence of pain and 10 indicating the worst imaginable pain. Distribution of the above variables between participants who accepted and those who declined the hypothesis of being recruited in a RCT were compared using the Fisher's exact test. To preserve a familywise type I error of 0.05, in accordance with Bonferroni principle a difference was regarded as statistically significant if *P* < 0.05/19 = 0.00263.

## Results

During the first semester of 2014 a total of 500 women were consecutively enrolled in the study. All the patients were assisted by the National Health Service and no incentives were offered to facilitate recruitment. No woman refused to participate in the survey. The mean age of the participants was 37 ± 8 years; 161 (32 %) were parous. A total of 354 (71 %) patients were using or had used medical treatments, whereas 301 (61 %) had undergone one or more surgical procedures. The diagnosis in the 199 women who did not undergo surgery was ovarian endometriomas (*n* = 95), rectovaginal endometriosis (*n* = 75), bladder detrusor endometriosis (*n* = 15), deep lesions infiltrating the pouch of Douglas and parametria (*n* = 10), and full-thickness bowel lesions (*n* =4). Non-surgical diagnosis was based on ultrasonographic criteria in patients with ovarian endometriomas [16, 17]; on visual inspection of the posterior fornix and biopsy of vaginal lesions in those with rectovaginal endometriosis [18]; on ultrasonographic criteria [19, 20], cystoscopic findings, and biopsy of vescical lesions in those with bladder detrusor endometriosis; on physical signs at recto-vaginal examination and ultrasonographic criteria [21–23] in those with deep infiltrating lesions; and on ultrasonographic criteria [24], and double contrast barium enema and rectosigmoidoscopy/colonoscopy findings in those with full-thickness bowel lesions. Magnetic resonance imaging was performed in selected circumstances. All the women compiled both questionnaires.

A total of 239 (48 %) women would decline participation in a comparative study on a new drug and a standard drug for endometriosis-associated pelvic pain, as 204 (41 %) would prefer the former medication, and 35 (7 %) the latter. Fifty women (10 %) would participate in a RCT, but only 24 of them (5 %) would accept blinding. The most frequently chosen option was the patient preference trial (211; 42 %; Table 1).

**Table 1:** Summary of women’s responses to questionnaires on willingness to participate in comparative studies on treatments for endometriosis

Women’s response	Number	%^a^
A. Willingness to participate in a study comparing a new and a standard drug		
No, because of preference for the new drug	204	41
No, because of preference for the standard drug	35	7
Yes, and would accept both randomization and blinding	24	5
Yes, but would accept only randomization and not blinding	26	5
Yes, but would like to choose the drug after information	211	42
B. Willingness to participate in a study comparing medical and surgical treatment		
No, because of preference for medical treatment	186	37
No, because of preference for surgical treatment	43	9
Yes, and would accept random allocation of treatments	11	2
Yes, but would like to choose treatment after information	260	52

^a^Figures are rounded to unity

A total of 229 women (46 %) would decline participation in a comparative study on medical versus surgical treatment for endometriosis-associated pelvic pain, as 186 (37 %) would prefer pharmacological therapy and 43 (9 %) a surgical procedure. Only 11 (2 %) women would participate in a RCT. More than half of the women (260; 52 %) chose the patient preference trial as their favorite option (Table 1).

We also compared the 50 women who would accept to be enrolled in a RCT on a new versus a standard drug, with the 450 who would decline participation in such a study (Table 2). The two subpopulations were substantially similar, without significant differences. We did not perform an analogous analysis for the questionnaire on the study on medical versus surgical treatment, as the group of accepters includes only 11 women, thus impeding any meaningful conclusion.

**Table 2:** Characteristics of women accepting or declining to participate in a RCT comparing two different drugs

Characteristics	Accepting RCT	Declining RCT	*P*	
	*n* = 50	*n* = 450		
Age (years)	39.4 ± 7.3	37.3 ± 7.8	0.07	
Previous deliveries	22 (44 %)	139 (31 %)	0.08	
Smoking	3 (6 %)	93 (21 %)	0.013	
BMI (Kg/m^2^)	22.6 ± 1.5	23.0 ± 2.5	0.31	
Education			0.45	
≤ 13 years	25 (50 %)	196 (44 %)		
> 13 years	25 (50 %)	254 (56 %)		
Previous surgery for endometriosis			0.59	
None	19 (38 %)	180 (40 %)		
One	24 (48 %)	186 (41 %)		
≥ 2	7 (14 %)	84 (19 %)		
Previous/current medical treatments^a^	35 (70 %)	319 (71 %)	0.87	
Oral Contraceptives	21 (42 %)	193 (43 %)	1.00	
Progestins	13 (26 %)	150 (33 %)	0.34	
GnRH agonists	1 (2 %)	20 (4 %)	0.71	
Non-conventional drugs	6 (12 %)	19 (4 %)	0.03	
Dysmenorrhea				
Biberoglu-Behrman grade 2-3	21 (42 %)	185 (41 %)	1.00	
NRS ≥ 8	14 (28 %)	149 (33 %)	0.53	
Dyspareunia				
Biberoglu-Behrman grade 2-3	9 (18 %)	91 (20 %)	0.85	
NRS ≥ 8	4 (8 %)	53 (12 %)	0.64	
Chronic pelvic pain				
Biberoglu-Behrman grade 2-3	7 (14 %)	77 (17 %)	0.69	
NRS ≥ 8	2 (4 %)	40 (9 %)	0.42	
Use of analgesics	29 (58 %)	240 (53 %)	0.55	
Number of days per month^b^	3.9 ± 3.7	4.0 ± 3.3	0.93	

^a^The sum does not add up to the total because some women had used more than one medication

^b^Refers to those who use analgesics

In order to assess whether a previous surgical procedure could influence the women’s attitude toward participation in a RCT, data were analyzed also according to surgical or non-surgical diagnosis, but no significant variations in distributions of answers were observed (Table 2).

## Discussion

The scenario emerging from this survey investigation conducted on a study population of Italian women with endometriosis, poses fundamental questions to clinical investigators in this specific area of research. In fact, only a small minority of patients would accept enrollment in a RCT on the treatment of pelvic pain. If this situation is not limited to our referral centre, generalizability of results of trials might be prevented even when planning multicentre studies in order to overcome difficulties in recruitment. In fact, the external validity of trials conducted on extremely selected study groups would be low, limiting the possibility of extrapolating findings to a broader, heterogeneous population.

Moreover, a comparison between medical and surgical therapy would be practically unfeasible, thus confirming that patients are not prone to be casually allocated to very different options [25–30]. This could potentially further distort the evidence, as only selected RCTs could be conducted (i.e., between two different medical treatments, but not between a medical and a surgical treatment), thus preventing complete assessment of all available interventions, as previously suggested [31].

Women appear nowadays much better informed than in the past, and wish to discuss treatment alternatives and to choose the option that best fits with their personal needs and expectations. In a sense, randomization impedes this tailored choice. This is confirmed by the fact that the most frequently selected option on both questionnaires was the patient preference trial. In other words, only some patients seem to be still willing to participate in clinical research and, in most cases, at their conditions, not at investigators’ conditions. In particular, 95 % of participants in our survey would decline blinding.

A potential drawback of our study lies in the characteristics of our participants, who are self-referred from the entire country, and sometimes have already undergone unsuccessful medical treatments or surgical procedures. Possibly, they might have a higher than usual level of knowledge on their disease, and of information on centers of expertise. It could be argued that this type of survey should be conducted only on women who have never been previously treated medically or surgically. Yet, this collides with the possibility of obtaining a definite diagnosis in most patients. Moreover, a laparoscopy performed with diagnostic purposes, eventually means treating that patient surgically in case endometriosis is found, as it would be unethical to visually confirm endometriosis without excising the lesions. In addition, the pattern of answers was not different in the 301 women who had already undergone a surgical procedure and the 199 who were never operated, thus questioning the impact of previous treatment on the study outcome. Finally, a misdiagnosis bias in the latter group appears highly unlikely, as the diagnosis of endometriosis was based on robust clinical, ultrasonographic, and histological evidence [32]. As study participants were all assisted by the National Health Service, a potentially spurious association between socioeconomic status and propensity toward participation in clinical research can be safely ruled out. More in general, clinical investigation is usually conducted in tertiary care centers thus this appear to be the type of population that would be considered for recruitment in a RCT anyway.

The groups of women that would accept or decline randomization in a trial on medical treatment did not differ significantly. However, our study was not originally powered to investigate the distribution of baseline characteristics between the two subgroups of women. Indeed, the number of participants was large and women were recruited consecutively, thus limiting the possibility of selection bias. Patients were carefully instructed about the different types of experimentation, on the objective of the present survey, and on the hypothetical nature of our inquiry, which would not have implied future obligations. They were allowed to ask questions and obtain clarifications by dedicated medical personnel. In addition, the compilation of the two questionnaires was very simple and could be performed rapidly. This helped avoiding refusals to participate in the survey.

Women could not select more than one option. This can be considered as another weakness of our survey and may explain why the patient preference trial was the most popular choice. Allowing women to choose more than one study design option, and structuring questions in a less “leading” format, would have probably helped clarifying whether randomization was absolutely unacceptable or would be a possibility.

Several investigators recently focused on the growing barriers to the conduct of RCTs, describing in details the types of impediments and suggesting different modalities to overcome unnecessary obstacles [5–8]. On the other hand, estimates of effect size in large observational studies may be precise, but remain substantially weakened by bias and confounding that only random allocation can control [8]. Indeed, the problem here is different: in spite of all shareable methodological considerations, RCTs in the endometriosis area appear increasingly difficult to conduct, at least in Italy, specifically because the vast majority of patients would decline random allocation.

The adoption of the partially randomized patient preference trial design, in which participants who do not accept randomization are allowed to chose their preferred intervention [33, 34] or of the response-adaptive randomization trial design, in which the ratio of participants assigned to each arm are actively adjusted in favor of the better performing intervention based on already available data of patients previously recruited [35], does not seem to overcome the basic issue of unwillingness to accept randomization. In any case, the resulting group of women allocated by chance would be too small to be representative of the population of women with endometriosis. Moreover, the administrative burden and the costs would be substantially the same as for the standard two-arm clinical trial design.

To complete their studies, several investigators conducted part of the trial based on random allocation of treatments, and part based on patient preference [25, 26, 29, 36]. Interestingly, more patients generally chose the patient preference option than the random allocation one. Moreover, both the estimates of effect size and its precision were substantially similar in the two study populations [25, 26, 29, 36]. Additionally, it has been reported that the results of well-designed observational studies are generally similar to those of formal RCTs [37, 38]. Therefore, when preferences based on informed expectations exist, observational methods may be an alternative to RCTs [39, 40].

When the objective is the comparison of already approved treatments for endometriosis (i.e., phase IV studies, thus excluding truly novel, experimental interventions), observational studies are easier and less costly to conduct than RCTs, can be carried out in “real world” patient populations, and can be prolonged for longer periods on a large number of participants, thus providing robust evidence on safety of interventions [2]. As treatments are not allocated randomly, multivariable statistical models must be adopted to control for potential bias and confounding factors. The physician’s selection of patients who should receive the treatment constitutes one of the main confounders in observational studies. Therefore, it is important to try to limit this bias. Considering the two hypothetical comparisons of our survey, two different study designs could be adopted when RCTs appear unfeasible.

When large differences exist in the type of treatments to be compared and in associated morbidity (in our survey, medical versus surgical treatment), the patient preference trial might constitute a practical alternative. Differently from an observational study comparing two case series, where clinicians allocate treatments, in the patient preference, parallel cohort trial, treatment allocation is by patient choice. Whereas clinicians may be prone to recruit different types of women to the two study arms, allocation by the patients themselves should improve external validity. It has been suggested that preference-based treatment allocation may optimize cost-effectiveness of intervention [41], also because this research environment may be more similar to practical life conditions [33]. In case of functional outcomes, a comparison between two groups of participants who have chosen their treatment emphasizes patient satisfaction, thus representing the maximum possible effect size of the intervention [42]. However, a major selection bias is introduced in a study based completely on patient preference, thus limiting the interpretation of the findings. Moreover, the effect observed under these conditions can be referred exclusively to patients who specifically choose that treatment [43].

When two similar treatments are being compared (e.g., a new versus an old progestin, or a progestin versus a combined oral contraceptive), the patient-preference trial design does not seem suitable, as women may not be able to clearly express a definite preference. In these cases, the before and after study (or pre-post study), a quasi-experimental design, may be an option [44]. The before and after study is usually adopted at a system level (clinics, hospitals) to compare outcomes before and after an intervention is implemented [45]. A before and after study design could be used also to evaluate treatments, provided some conditions are satisfied. In this case all new cases of patients with symptomatic endometriosis would receive the same, standard medical therapy for a pre-planned period of time, at the end of which all new cases with the same clinical characteristics would receive the new medical treatment for the same period of time. The study gauges the difference in the effect of the new drug compared with that of the standard one.

In order to infer that a variation in outcomes is the consequence of the implementation of the new treatment, the characteristics of the participants in the “before group” must be similar to those in the “after group”, otherwise such inference would be seriously flawed. In order to avoid a selection bias, all eligible patients observed before the introduction of the new intervention must be included in the “before group”, the new treatment must be implemented at a precise time point, and all eligible patients observed after that cut-off time must use only the new treatment and be included in the “after group”. Any admixture of different treatments during the study period would invalidate the findings. There should also be no evidence of a prevailing temporal trend. Still, without a control group of patients in whom no variation in the intervention has been implemented, it may reveal difficult to ascribe differences in outcomes to the change of medical treatment. Moreover, only random allocation of treatments allows genuine comparability of the study groups. In a before and after study, even a modification in referral pattern during the study period may result in the creation of populations that may differ for several characteristics, such as severity of symptoms or type of lesions. Since it is unlikely to be able tocontrol for all known and unknown characteristics that may influence the outcome, it may not be possible to exclude that any observed between-group difference in a before and after study is due to confounding [45].

## Conclusion

The surprising findings of this survey should be considered as an impediment that our community must try to overcome. Indeed, the quality of the evidence derived from observational studies remains suboptimal even under the best research conditions. In particular, generalizability of results of patient preference trials is limited, and inferences from pre–post studies should be regarded as being based on circumstantial evidence. More in general, observational studies may be viewed as a mean to investigate whether interventions that have already been proven to work under ideal circumstances (that is, in RCTs), work also in non-selected populations, and which treatment alternative works best in real life. In other words, observational studies can be used to evaluate *efficiency*, although they are not suited to assess *efficacy*, that is whether a new, experimental treatment can work. Therefore, observational studies may be useful for defining the role of the available therapeutic armamentarium in different everyday clinical conditions. However, experimental treatments that could interfere with the pathogenic mechanism(s) of endometriosis are indeed needed. Thus, the conduction of RCTs remains a priority.

Different strategies could be adopted to make patients understand that participating in RCTs is in the best interest of all women with the disease. The engagement of patients could be increased by including them in the selection of therapies, design of protocol, recruitment, and writing of lay summaries and scientific articles [46–50]. According to some authors [51], patients have a greater ability, compared with clinicians, to prioritize the outcomes they truly value, identify obstacles to trial enrolment, and help discover what works in the real world, not just in research settings. Moreover, health authorities and scientific societies should increase their educational initiatives toward citizens, clarifying the advantages of well-conducted clinical investigation and explaining the benefit for the entire population that derives from it. In this regard, patients associations could play a crucial role, disseminating factual information and encouraging participation in trials. In the end, accepting enrolment in a RCT is an act of generosity that entails a high sense of social responsibility.
